# Application of Fuzzy Composite Programming in a Questionnaire as a Methodological Test to Study the Effect of Reservoir Management on Social Interests—A Survey Based on Two Case Studies in Southern Germany

**DOI:** 10.1007/s00267-023-01799-9

**Published:** 2023-02-11

**Authors:** Milan Daus, Daniel Weber, Rüdiger Glaser

**Affiliations:** 1 Physical Geography, University of Freiburg, 79085 Freiburg im Breisgau, Germany; 2 Nassauische Heimstätte, Schaumainkai 47, 60596 Frankfurt am Main, Germany

**Keywords:** Water management, Reservoir management, Social impacts, Fuzzy composite programming, Conceptualizing social implications, Compromise solutions

## Abstract

To understand the concerns, approvals and disapprovals of expert opinions about managerial issues from around reservoirs this study uses the approach of Fuzzy Composite Programming (FCP) in direct questionnaires to parameterize and rate a set of indicators with statements about managerial issues concerning societal implications by the responding experts. The personal ratings get summarized in four different layers and converted into one final numerical value which will be in the range of 0 as the absolute disapproval of the indicators and 1 as the absolute approval of the indicators. The FCP approach thereby rates the individual indicator, secondly the indicator category, thirdly the compensational factor and fourthly the dimensions of sustainability. This facilitates a rapid comparison of results of rather complicated sets of pre-set indicators in topics reaching from legal issues to societal concerns in one final numerical value to identify crucial topics and start open debates. This study was carried out as a methodological test at two water reservoirs in southern Germany. The results show a general possibility of using a rather retrospect methodology towards current ratings of experts in the field of reservoir management. 10 respondents answered the FCP questionnaires, 5 at each study site. The scores of the calculation showed a higher level of positive connection in the case of the Schwarzenbachtalsperre (SBT) with a score of 0.77, compared to a score of 0.54 in the case of the Franconian Lake District (FLD). Apart from the pure numerical scores, FCP can show conflicting issues and possible compromise solutions between the different stakeholders, in/based on the individual ratings. The findings could help reach a more sustainable management of water resources that includes all stakeholders, by pointing out debatable implications.

## Introduction

Water and energy are amongst the most important resources on earth being used by humans. The harnessing of energy derived from gravitational forces of water is an old technique, nevertheless, its use increased through technical advances and growing numbers of people living on earth (World Energy Council 2016), whereby dams are providing the major source of renewable electrical energy on a global scale (Sovacool and Walter 2018). The motivation to build the expensive and in size always growing dams consisted out of a multitude of reasons (Sivapalan and Blöschl 2017). Historically it was to provide drinking water, process water (for agriculture, business, and industry) and to secondly tackle the problem of flooding by controlling the tributary rivers in geologically suitable areas (Brüggemeier and Rommelspacher 1989; Föhl and Hamm 1985). To provide water for irrigation was and is one of the main reasons to build dams in drier regions of the planet, e.g., along the Nile (Strobl and Strobl 2011). The need for large amounts of electricity and freshwater resources led to an accelerating speed in building this kind of infrastructure during the 20th Century (Ansar et al. 2014; Awojobi and Jenkins 2015; Vorosmarty et al. (2004); World Commission on Dams 2000), a trend which is likely to continue in the course of the 21st Century (Zarfl et al. 2015) with large impacts on the environment, initializing the geologic epoch of “the Anthropocene” (Bai et al. 2016; Crutzen 2002; Crutzen and Stoermer 2000). Planning and integrating all interests into the management of large dams and reservoirs is of paramount importance (Linton 2014). Therefore, to gain knowledge of social perceptions of large infrastructure projects from a holistic and transdisciplinary point of view is of vital importance (Carr et al. 2020), in order to prevent negative consequences from early on (Faria et al. 2017).

A holistic approach is herein understood as integration of all stakeholders. This helps fostering social interaction and in the long-term possible acceptance and more importantly participation of renewable energy infrastructure by all stakeholders without compromising any interests (Tabi and Wüstenhagen 2017). It can enhance the participation processes in renewable energy projects and eventually lead to more benefits for the (local) communities (Gonzalez et al. 2020). A more holistic knowledge and participation can support a sustainable and integrated management framework for infrastructure such as hydroelectric dams and their reservoirs (Daus et al. 2021; Savenije and van der Zaag 2008). Therefore, this study uses a Fuzzy Composite Programming (FCP) methodology approach on data collected about social questions in the management of water resources in order to provide a set of parameters aiming at easing this challenge in hydrologic management. Social and societal questions however are harder, or impossible to grasp with distinct numerical values than e.g., technical issues. Opinions by experts in the field on selected statements are therefore transformed in FCP to numerical values on a scale from 0–1.

To find an optimal decision is a high aim in many disciplines from planning to the final construction in infrastructure projects. Multiple Criteria Decision Analysis (MCDA) provides methodological tools to find optimal solutions in terms of costs, hardware, operation, environmental impacts, and other factors. There are multiple options and methodological pathways to approach decision analysis in MCDA methods with different mathematical modeling to rank, select and compare different results (Cinelli et al. 2014; E. Roszkowska and Kacprzak 2016). The FCP approach roots in the MCDA methodological basis and enhances this evaluation by introducing factors to be rated personally by experts in the corresponding fields. Social aspects of planning, together with sustainability aspects, are becoming more and more important to be analyzed alongside planning infrastructure, especially since it has been largely ignored in the past (Kühne and Duttmann 2019).

Real-world questions are highly complex. So, the FCP approach could only account for some of them, to still be able to transform the results to real world managerial problems without exceeding a limit of complexness for the respondents (Haas and Stork 2016). The challenge herein is to find operationalizing indicators for the approach to account for the interconnectedness and complexity in the water-food-energy(-health)-nexus (Liu et al. 2017). Therefore, this paper aims to introduce the methodology of FCP (Andras Bárdossy et al. 1985; András Bárdossy and Duckstein 1992), mathematically based in fuzzy logic and fuzzy set theory (Carter et al. 2021; Esteban Indurain et al. 2018), by directly approaching people with expert knowledge on managerial aspects of (hydroelectric) reservoir systems in southern Germany to promote initial results about possible conflicting issues. Previous research on the perception and implications of reservoirs and dams use interview data (Daus et al. 2019; Piróg et al. 2019) and questionnaires (Dopico et al. 2022) and are mostly carried out at case study perspectives (Salinas et al. 2019). FCP may present a possibility to enhance the methodological options in this field.

While the methodology of FCP was usually used to rate indicators in respect to research questions by the researchers themselves, it is herein rated by individual respondents. Our methodological adaptations of the classical FCP represent a new approach in acquisition and evaluation of qualitative data sets, representing expert opinions and may introduce a new way of using an innovative method of empirical (qualitative) social research to obtain first impressions of perceptions and conflicts concerning water management.

## Materials and Methods

### Case Studies

**Fig. 1:**
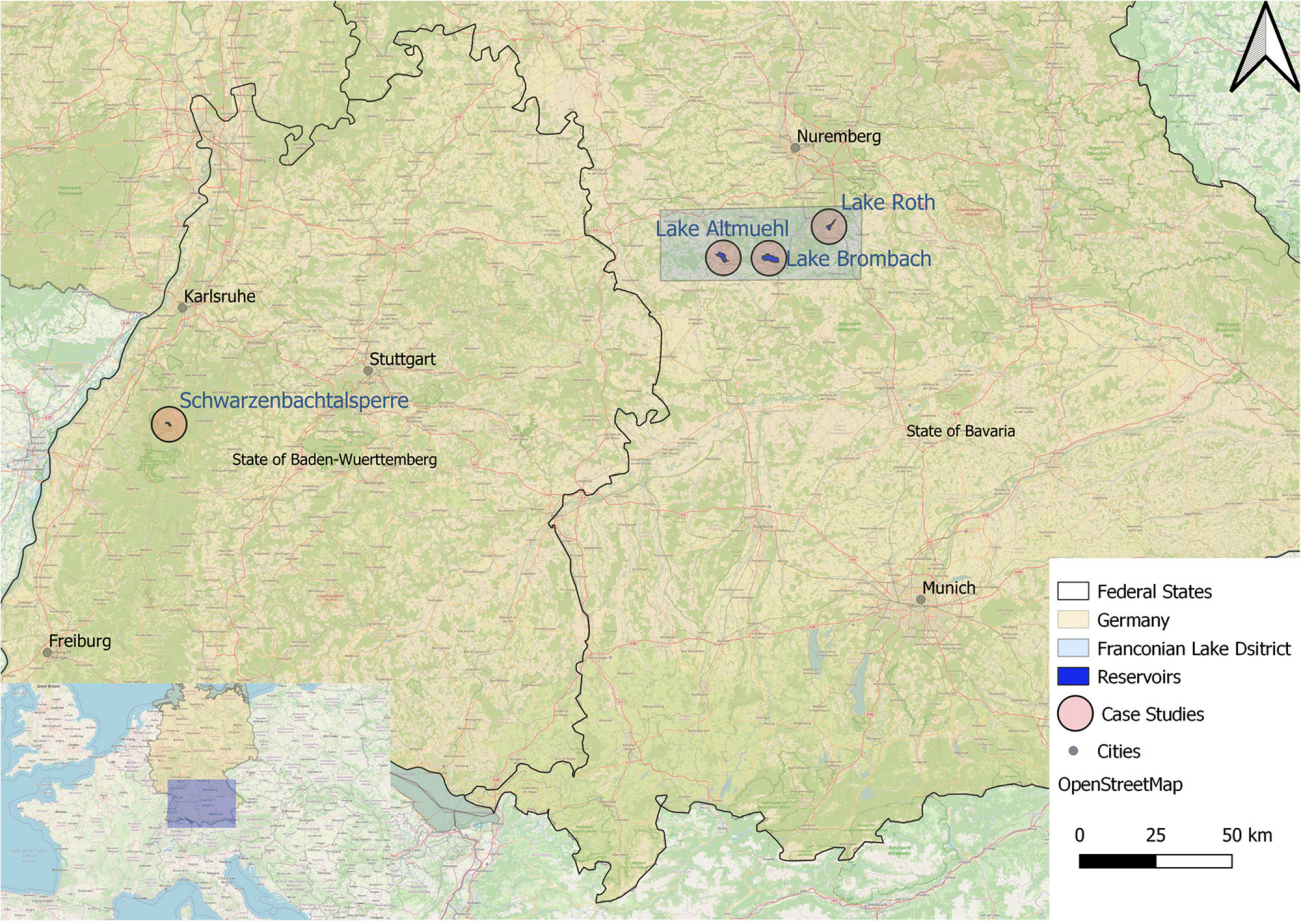
Overview of research area and case studies

The setup and interactions in human-environmental systems, like dams, are complex. In most cases, dams have more than one primary function such as hydro electricity production, flood control, drinking water and irrigation, influencing many different spheres, and vice versa being influenced by manifold subjects (Kirchherr and Charles 2016). The two reservoirs researched as case studies, the SBT (*at 48°39'28.2“N and 8°19'26.5“E*) in Baden-Wuerttemberg and the FLD (*at 49°07'57.3“N and 10°55'51.1“E*) in Bavaria, are no exception. They were chosen as case studies for their very individual environmental, social, and technological nature. See Fig. 1 for an overview.

Both case studies lie in geographically distinct areas. A brief description of their genesis and basic layout helps the reader understand their intensions, respectively the purposes of their construction and how they developed. The SBT can be counted to the older reservoirs in Germany. With the completion in 1926, representing an outstanding innovation and high technology development at that time, electricity was being introduced to the rural areas of the Black Forest (Janzing 2002). It is part of the Rudolf-Fettweis-Werk, a power station complex, consisting of different smaller dams and reservoirs to mainly use the SBT and the head of water to the lower lying Murg valley for the production of electrical energy in the town Forbach (Keller 2014). The Murg valley in the Black Forest with its bedrock of granite and bunter was always being used for gravitational water energy. If it was formerly the transport of wood with rafts of tree logs, it is now the production of energy with hydroelectric installations (ibid.).

The FLD is a comparatively new development. With a resolution of the Bavarian Parliament in 1970, it was decided to build a series of larger reservoirs to transfer water from the Altmuehl (Danube)-catchment to the Main (Rhine)-catchment. As the northern parts of the Bavarian state receive(d) much less precipitation and experienced difficulties concerning low water, which impeded navigation and industrial processes alongside and on the Main river (Briemle 1996). The FLD was finished more recently with the Large Lake Brombach being completely filled in the year 2000 (Köngeter 2013). The motivation to build the reservoirs was therefore different from the SBT and included comparably smaller hydro energy installations but larger touristic infrastructure.

Current developments mirror the ongoing debates and developments at the SBT and FLD on an almost daily basis. The planned and anticipated addition of an underground pumped storage at the SBT (Achatz and Kamuf 2016; EnBW 2018) to account for a larger revenue in electrical energy is a current topic. Additionally, plans to install large touristic infrastructure at FLD failed by a referendum with a small majority (52.4%) (Grun and Bayerischer Rundfunk 2021), which shows very vivid and critical involvement of stakeholders that form the course and development of these reservoirs.

The research in this study was carried out within the research project CHAllenges of Reservoir Management (CHARM; www.charm-bw.de), consisting of the three universities of Stuttgart, Konstanz and Freiburg and an interdisciplinary team with engineering, natural and social sciences combined to find answers to critical managerial questions concerning reservoirs. These questions encompassed current concerns from silting, over cyanobacteria blooms and social concerns reaching from legal issues, over perception to energy backbone functions of reservoirs.

### Water-Energy-Environment-Social-Nexus

Since the integration of different natural/social/managerial spheres will always bring up intertwined connections as the interactions are manifold, this study is only able to glimpse at the ones identified in the course of this work and preliminary studies (Daus et al. 2019; Daus et al. 2021). As the aim in this research was to find out more about managerial implications on social and societal issues in reservoir management, this complexity is tried to be covered through the research design. Managerial endeavors of water projects always have been of great importance. Even though the primary objectives in constructing water projects have shifted in the course of history, management of these infrastructure projects gained in importance, as did the complexity in the systems they are built in. Historically, technical solutions were favored to challenges from an engineering science perspective. This approach gained urgency with increasing population and industrial and agricultural production in the 20th century (Pahl-Wostl 2015). In the present day, questions about managing those large infrastructure projects emerge on very different scales, including more and more social and environmental aspects (Pahl-Wostl 2015). This fact emphasizes a deeper understanding of the nexus in the management of reservoirs and dams, which is tried to be enhanced through the application of a new methodological approach.

### Fuzzy (Linguistic) Conditions Demand Fuzzy Set Methodology

Social acceptance is a widely used term in the field of policy and technology development, but it is hardly ever defined specifically and objectively (Wüstenhagen et al. 2007). Participation may be a better solution to the acceptance concept. Therefore, to get an idea of the social conceptualization and acceptance/participation, this study tries to shed light on the term acceptance/participation and conceptualization by using the methodology of FCP (Bardossy et al. 1993; Andras Bárdossy et al. 1985; András Bárdossy and Duckstein 1992; Bàrdossy et al. 1984; Bogardi et al. 1983) in researching reservoir systems and thereby giving examples on how perception may be a more suitable term to describe the manifold connections within a social network around artificial reservoir lakes. For the first time, this is undertaken by direct questioning with FCP of experts in the field.

The expert function is a distinct form of expertise, where the respondent is chosen because of their professional function and not as a person itself. The sample is, other than in the quantitative methodology, independent from a specific number of participants. It is important to be able to generalize the findings of the questioning and relate them to similar examples and other affected parts of socio-economically embedded systems (Mayer 2013).

Indicators to be rated were stating managerial issues at the corresponding case study. The topics encompassed implications found in previous studies, reaching from legal issues to case specific problems with e.g., cyanobacteria, fluctuating water levels and conflicts between different uses (Daus et al. 2019; Daus et al. 2021). These issues were translated into indicators to be rated by the respondents, e.g., “Different interests are considered during operation”; “Uses other than water management are often restricted”, “There is fairness and willingness to talk about all current developments concerning water management”. The value of 0 would represent no approval to these indicators, whereas a rating of 10 would mean a complete consent.

The FCP approach gained in popularity within the field of water based environmental decision-making research and its effects in the last decades of the 20th Century. However, the use of FCP is still scarce. Bogardi et al. (1983) used FCP to approach a watershed in western Hungary facing bauxite mining effects in a karstic aquifer. Bàrdossy et al. (1984) researched FCP in connection to a Hungarian and Austrian regional water management plan. Lee et al. (1991) researched effects in managerial questions about contaminated dredged materials using FCP. Jones and Barnes (2000) used the method to find decision support in precision farming by using remote sensing and crop model data. Freeman (2008) used FCP to modernization criteria for water resources planning. Haas and Stork (2016) tried to bridge the gap of natural/engineering sciences and social sciences by researching social/environmental conflicts of water management in flood control projects.

### Challenges and Methodological Adaptations for the Purpose in This Study

**Fig. 2:**
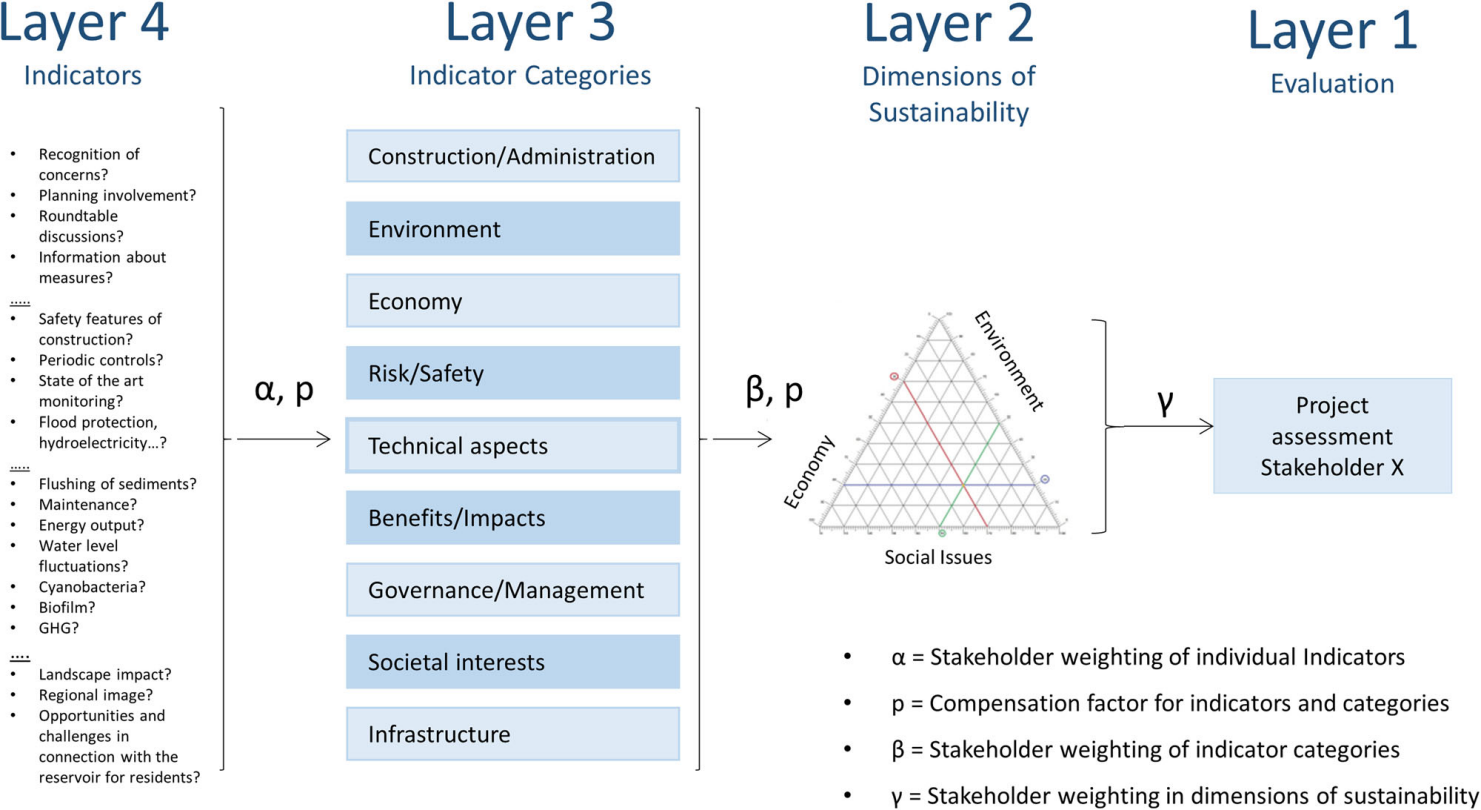
Generalized workflow of FCP approach with example indicators, modified according to Haas & Stork (2016, p. 90)

The need to adapt the methodological approach became apparent since the focus of rating was based on retrospective rating and weighting by the researchers. In this case, we wanted to compute a questionnaire rated by respondents directly. Therefore, a few adaptations were needed (see Fig. 2), which are explained in the next section.

- α-factor: Percentage ratio of the weighting of all individual indicators per indicator category.
- β-factor: Percentage ratio of the weighting of the indicator categories to each other.
- p-factor: Individual weighting of the indicators and indicator categories (as β- p-factor).
- γ-factor: Percentage ratio of the weighting of the three dimensions of sustainability to each other.

Our modified version of FCP uses four layers that compute different aspects of managerial issues around the case studies:

- Layer four represents the statements (so-called indicators) that are to be rated and weighted individually by the respondents.
- These are then summarized and weighted into nine respondent indicator categories, in this case here 1: Administration and Construction, 2: Ecology/Environment, 3: Economy/Economic Issues, 4: Risk and Safety of the Reservoir, 5: Technical Issues, 6: Benefits of the Reservoir, 7: Governance/Management, 8: Social Interests, 9: Infrastructure (Layer three).
- Each indicator category is then weighted with a compensational factor when the respondent thinks of it to be especially important (Layer two).
- Finally weighted into the dimensions of sustainability (in between Layer two and Layer one) to get a final numerical value between 0–1 (Layer one) (see Fig. 2).

The total score of 0 is representing a total disapproval of the indicators in question and the total score of 1 is a complete approval. The indicators can thereby have a positive or negative prefix depending on their linguistic meaning. E.g. if a negative meaning is associated with an indicator, the prefix will be negative and vice versa.

The FCP methodology represents an approach that intentionally provides possibilities for respondents to rate the indicators personally and therefore accounting for subjective preferences. With the final mean value, it is possible to identify conflicting issues and compromise solutions in Layers three & four. This means that - in contrast to other MCDA methods—the final scores can be interpreted not only by their actual numerical value but more importantly by the individual ratings of the respondents to account for differences and similarities between the stakeholder ratings. The indicators thereby target very practical issues for the respondents arising within and around the reservoir projects and their management. The single indicators are summarized into indicator categories, facilitating a quicker overview and more possibilities of individual ratings, which are explained in the course of this chapter.

To conduct this survey a pre-test of FCP developed methodological optimizations for the purpose of this study and was undertaken by the authors in a preliminary study (Weber 2016). The pre-test showed the necessity of adaptations in the mathematical model. Especially in cases where not every indicator would get rated by the respondents, the model needed some adaptations in certain parameters.

The conceptualization and methodological measurements of social, economic, and ecological impacts of infrastructure are complex, with myriad impacts occurring over dimensions such as time, space, societal values, and perceptions (Kirchherr and Charles 2016). The herein proposed new approach of using FCP is used to account for uncertainty within the indicators and integration of sustainability spheres (social, environment, economy), considering the assessments of expert knowledge (Arunraj and Maiti 2009). Fuzzy set theory can account for the vagueness in language we use to describe any contexts of our world. E.g. the personal assessment “somewhat” or “a little bit”, which also applies to social views and values on water reservoir managerial questions (Carter et al. 2021; Zadeh 1965). The analysis of FCP indicators is of course biased in the use of linguistic descriptions to our environment.

This FCP approach worked with the “total weighting approach” (Greco et al. 2016; Polatidis et al. 2006), where the weight is calculated with one absolute numerical value. In this case, the weight and numerical value was either in the range of 0–10 (and weighted with the α-factor), Important-unimportant (*β*-factor and factor *p*) and relative to the other two dimensions of sustainability (factor γ) (see Figs. 2 and 4). This approach has its roots in utility-based-models. Standardization-options for a complete mathematical compensation possibility facilitate a relative ratio of the weightings to each other, depending on the individual ratings of the factors (Wilkens 2012).

With weighting of the different levels of layers the FCP approach facilitates deeper personal and professional weightings of indicators, indicator categories and dimensions of sustainability in layer four and three with the α (alpha) factor (see Figs. 2 and 4) and the factor *p* for (in)compensability of indicators and categories.

Each individual indicator rated with a number between 0 and 10 can be weighted by the respondent with the α (alpha) factor. An average rating is assigned the number 1. This value is doubled for each increase in weighting, and the value of the weighting factor is halved for a decrease in importance. Accordingly, an individual indicator classified as “irrelevant” has a weighting factor of 0.25, four times less than an indicator with an average weighting; in return, a criterion classified as “very important” is multiplied by a factor of 4. Once a scale has been defined for determining the weighting factor, the next step is to normalize the assigned values:

$$w_j = \frac{{g_j}}{{\mathop {\sum}\nolimits_{i = 1}^n {g_i} }}$$

*w*_*j*_ = *normalized weighting factor for the indicator*; *jg*_*j*_ = *non-normalized weighting factor for the indicator*; *ji* = *Indicators* 1, 2, 3…*n*

The individual scores of differences in indicators and indicator categories get exponentiated in their percental share of the final numerical value through the so-called compensation factor *p* for indicators and their indicator category. This means, in addition to the single indicators, the respondents are able to increase/ attenuate the corresponding indicator categories, in this case 9 categories, accordingly. The rating will enhance the share of the indicator and its category in the final numerical value. This step will be executed by the *β* (beta)-factor in connection with *p*. With the *β* − *p* (beta)-factor the indicator categories get weighted as follows:

$$\beta _p = \frac{{p_i}}{{\mathop {\sum}\nolimits_{i = 1}^n {p_i} }} \cdot \left( {1 - \beta _i} \right) + \beta _i$$

*β*_*p*_ = value of indicator category in the score of a dimension of sustainability; *n* = number of indicator categories; *p*_*i*_ = indicator category; *β*_*i*_ = *β* factor of the indicator category

**Fig. 3:**
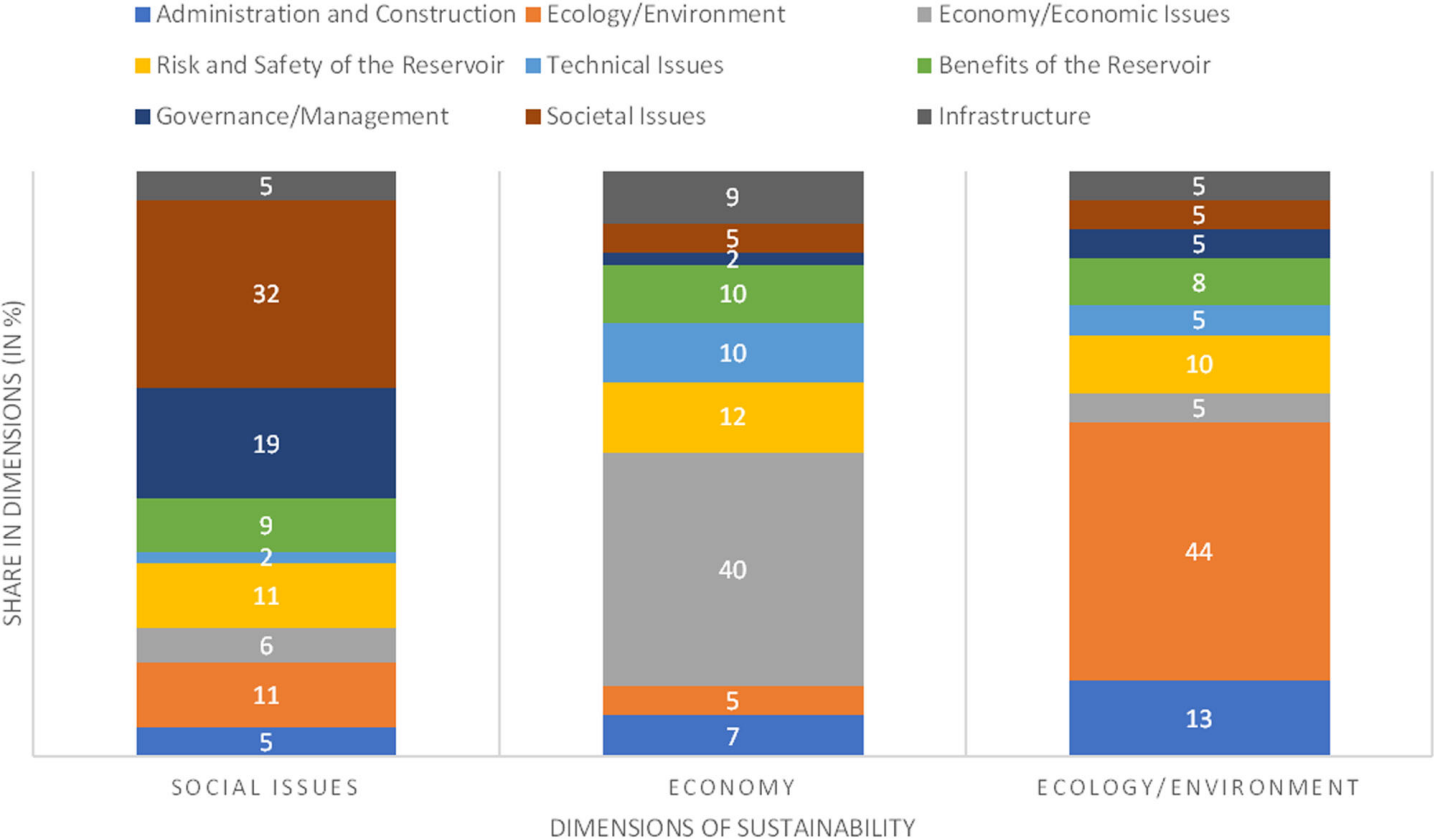
β-factor (indicator categories) percentage shares in dimensions of sustainability, modified after Haas and Stork (2016, p. 95)

The compensational factor therefore emphasized, or reduced the calculated numerical values in Layer two again. A final step allows the respondents to set a score in the dimensions of sustainability. To provide a pre-set list of shares of each indicator category in the sustainability dimensions defined by a delphi-approach (Barrow 2010; Haas and Stork 2016; Green et al. 2007) questioning of a group of reservoir management experts (from engineering, environmental and social science) about the shares of the indicator categories in the three dimensions: society, environment, economy (See Fig. 3).

One challenge in this particular approach using FCP in direct posed questionnaires is the potential appearance of gaps in the weighting and rating of indicators by the respondents. Since the questions demand a certain knowledge, it is likely that not every single indicator can be rated and therefore gaps will occur in the data set. This process can be accounted for with the so called “gap factor”, which attenuates the numerical value of gaps with rising occurrence. This factor accounts for unrated indicators and indicator categories:

$$I_k = \frac{{\left[ {\left( {1 - \mathop {\sum}\nolimits_{i = 1}^n {x_i \cdot \alpha _i} } \right) \cdot q} \right]}}{n} \cdot l + \mathop {\sum}\limits_{i = 1}^n {x_i \cdot \alpha _i}$$

*I*_*k*_ = total weighting of indicator category; *kn* = number of indicators; *x*_*i*_ = rating of indicator; *xα*_*i*_ = weighting factor for indicator; *xl* = number of gaps in indicator category; *kq* = threshold between 0 and 1

Methodological the γ-factor (between Layer two and Layer one) is a question of ratio in the three dimensions of sustainability, namely social issues, economy, ecology/environment. By default, the ratio is set to an equal 33.3‾%. The respondents have the opportunity to adjust the proportion after their personal preference towards the corresponding dimensions of sustainability by changing the default value of 1.

### Creating a Questionnaire and Finding Respondents in the Two Study Areas

**Fig. 4:**
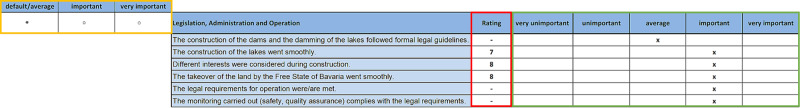
Impression of the FCP questionnaire of FLD in Microsoft Excel with -, - and  -factor ratings

As the methodological process of rating and weighting different indicators, indicator categories, compensational factors and dimensions of sustainability is complex, the aim was to find respondents who possess enough expertise to be able to rate and weight most indicators. This led towards a snowball technique to find suitable respondents in the study area(s). The interviewees herein were recruited through a snowball approach in previous studies using interviews (Daus et al. 2019). The experts involved in this study were guaranteed anonymity. Therefore, it is only possible to give information about the field of expertise and some general data. From the 10 participants, 2 were female and 8 were male. The questionnaire, programmed in Microsoft Excel (2019), was sent out via e-mail to 14 potential respondents at FLD and 9 at SBT, who have a background in reservoir management, general administration, environmental protection, touristic and cultural administration, agricultural and forestry administration, energy production, and associations in fishery and forestry professions (see Fig. 4). At both reservoirs five questionnaires were send back to the authors for evaluation. In summary, the respondents were confronted with a questionnaire containing 103 indicators at FLD and 88 indicators at SBT to be rated. For data protection reasons, the ratings are assessed with aliases e.g., SBT 1, FLD 1, etc.

A version of the questionnaire is available online in the data availability section. The final score is converted into a score between 0 and 1, where 0 is the absolute negative outcome and 1 the optimal stage. Again, it is not only the distance of the final result to an optimal point, but more the individual distances of the different ratings, that can show possible agreements and conflicting points.

### Data Gathering with FCP Questionnaire

Before the numerical analysis is illustrated, the practical workflow of this endeavor is a crucial part in this section. As this methodological approach was mostly used in a retrospect way and on rather objective scales as in technical and environmental ratings, the direct use of a questionnaire was unexplored. At first, it is to say that the respondents indeed worked with the questionnaire and filled out and rated the indicators intuitively despite its complex nature. Some of the pre-chosen indicators were enhanced with deeper insights by the respondents. With the target group of experts, this was to be expected since the researchers only have limited insight into the matter themselves.

The return rate of 0.36 at FLD and 0.55 at SBT of questionnaires in the target group of experts shows a relatively good feedback, in face of the time-consuming effort and the unattributed approach via e-mail. As the group of respondents was rather small overall, the willingness to answer an extensive questionnaire was impressive and may point towards a high need for starting debates about social implications of reservoir management.

The questionnaire was designed in a spreadsheet calculator for general usability. An instructional manual was sent out with every questionnaire. The rating and answering however required getting used to and could be counter-intuitive at first glance. This fact indeed reduced the intuitive use of the questionnaire, even though the instructional manual picked it up as the central theme. On the other hand, a dropdown choice for ratings and simple clickable marks to set the desired individual weights simplified the use of the questionnaire.

The respondents were all contacted via e-mail which allowed an equal treatment and no interference by the researcher. On the other hand, this fact prevented the respondents to pose direct comprehension questions that exceeded the explanations in the instructional texts.

## Results

The results in the evaluation of this approach showed that in a relatively small sample size, the direct questioned FCP approach produces well-arranged results in one final numerical value, without compromising any information on how these values were generated. The mathematical approach does indeed work, with improvements and lacking issues that are discussed in “Discussion”. Furthermore, this study could show the general possibility of confronting experts in the field with explicit indicators about aspects of managing reservoirs and reservoir systems to be rated and weighted individually. The evaluated FCP questionnaires showed aspects of individual and global assessments of the respondents in a summarized manner.

**Fig. 5:**
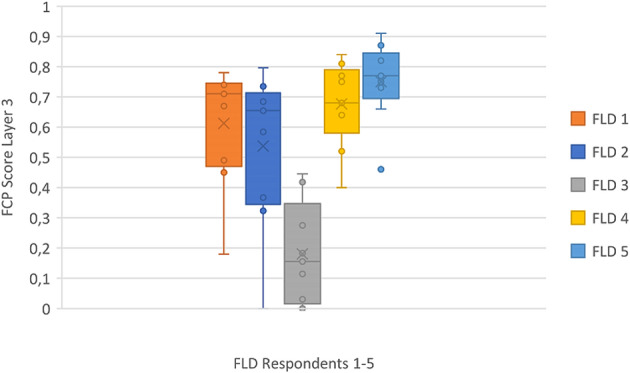
FCP Layer three scores of FLD study area (anonymized), with the horizontal line being the median and x the arithmetic mean

The questionnaires showed variability throughout the data set both across as well as within the study areas. Since the results of Layer four are summarized in Layer three, a distinct summary of Layer four is excluded graphically here for clarity reasons (see supplementary materials for details). Figure 5 shows the results of Layer three of the FCP approach in the FLD. The comparably heterogeneous ratings in this set reveal contested topics, with respondent *FLD 3* being the lowest outlier and *FLD 5* the highest. The average scores thereby vary quite heavily. The categories ratings represent the summary of the individual assessments of the indicators, plus a compensational factor to boost or decrease the scores. The categories were rated as follows with the extreme values reaching from 0 to 0.91 in the data set.

**Fig. 6:**
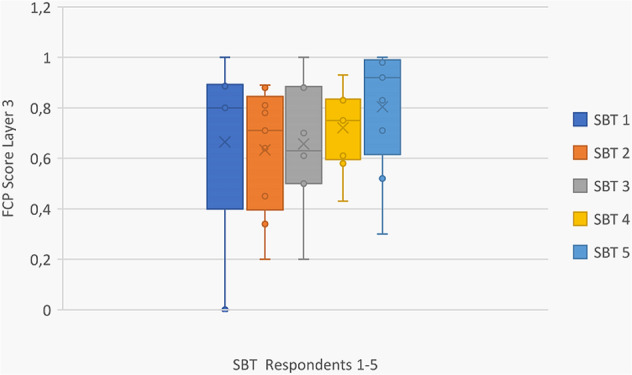
FCP Layer three scores of SBT study area (anonymized), with the horizontal line being the median and x the arithmetic mean

At the SBT the results showed a slightly different score with a tendency to higher scores overall in layer three which corresponds to higher ratings in layer four. The respondents’ ratings in SBT have a clear tendency to be more positive or affirmative than in the FLD, creating a more homogeneous field of individual scores (see Fig. 6). The ratings were less critical, or paraphrased more positive, which offered more potential for compromise management solutions.

**Fig. 7:**
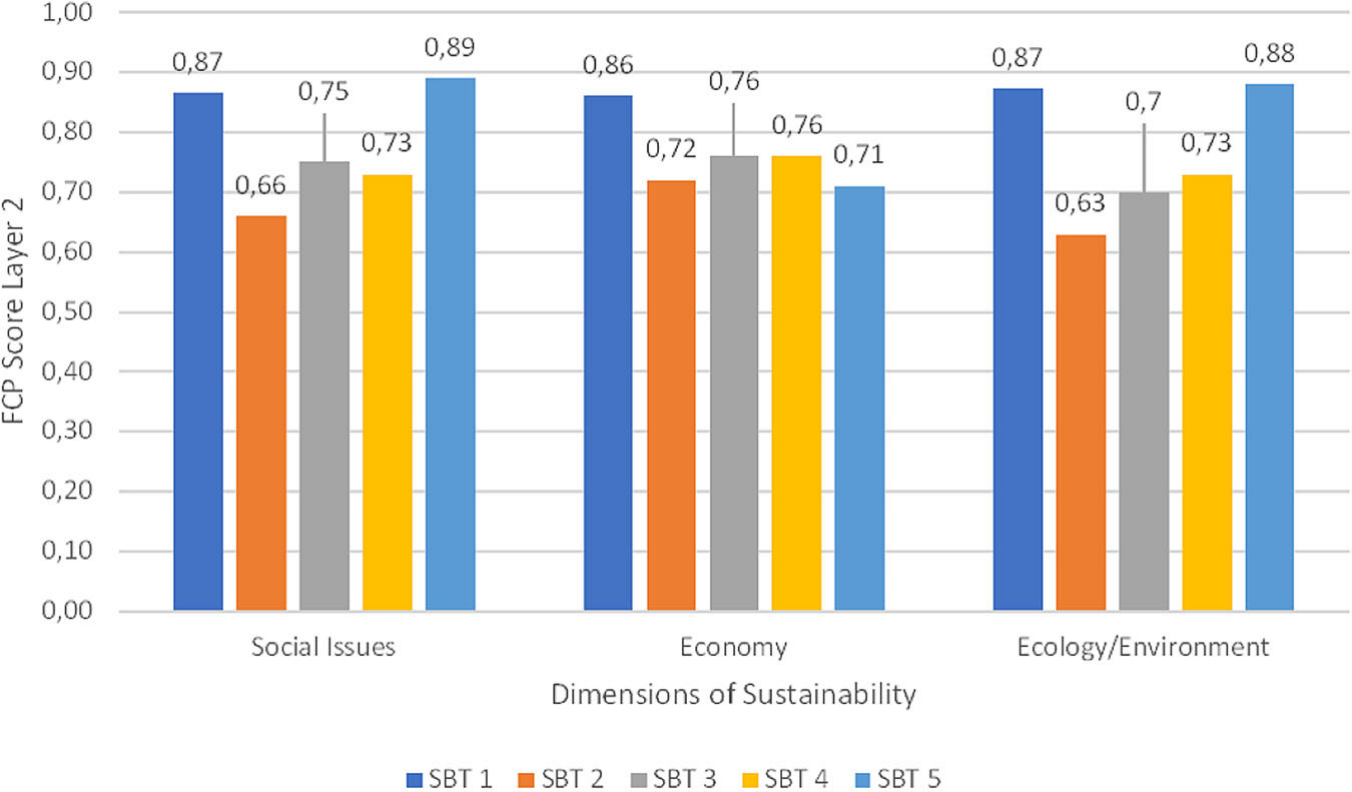
FCP layer 2 scores with shares of each dimension in individual questionnaires at SBT

**Fig. 8:**
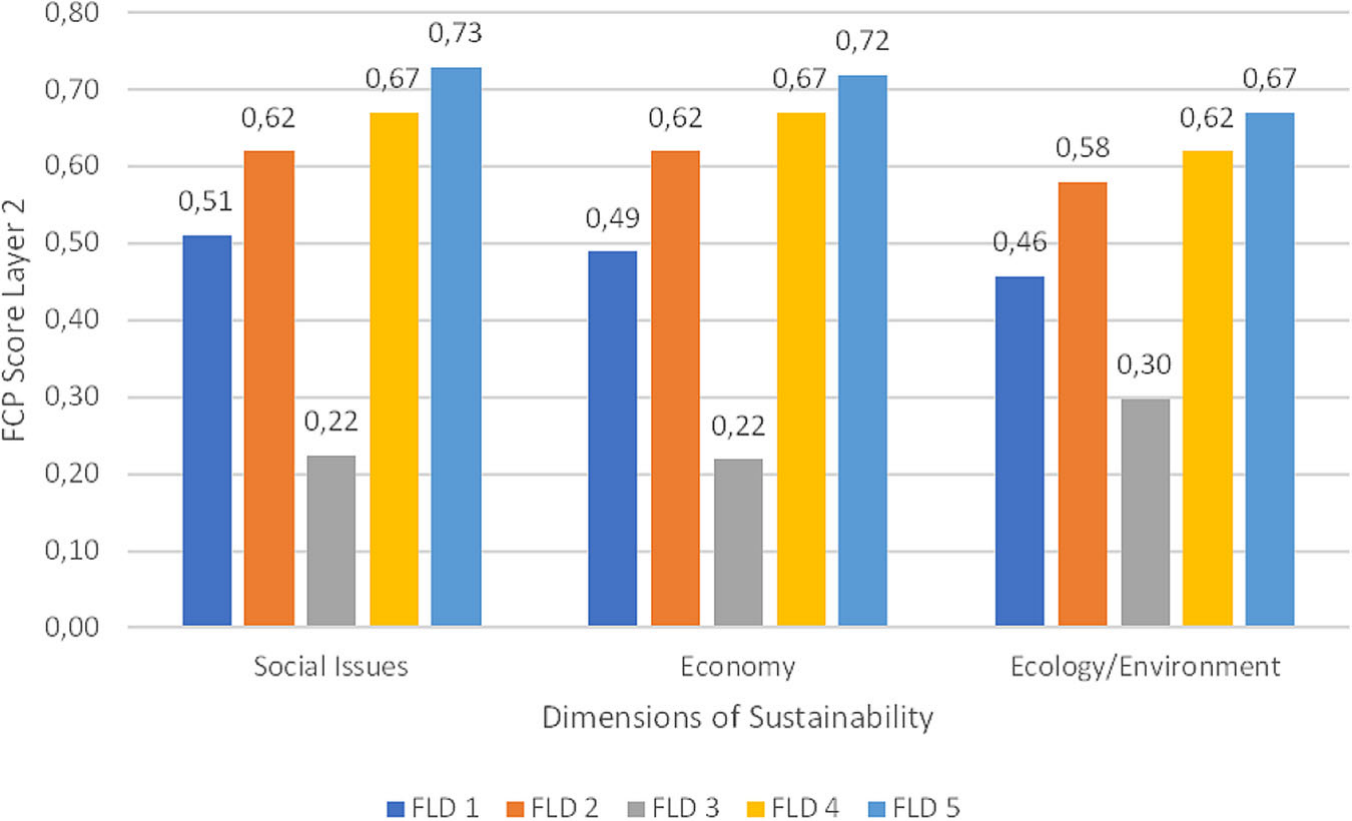
FCP layer 2 scores with shares of each dimension in individual questionnaires at FLD

If we take a look at the layer two of the FCP approach, the dimensions of sustainability show different aspects of the individual tendency with the summary of the shares in the indicator categories shown in Fig. 3. Figure 7 displays the scores of the γ-factor at the SBT. Figure 7 offers a possibility to view personalized ratings once more with the inclusion of the γ-factor for the aggregated layer 2 results. In the final step, these numerical values get aggregated to one final score. The respondents increased the impact of the dimension of ecology/environment in two cases by tripling it to 60% (SBT 1) or doubling it to 50% (SBT 2). The other three respondents kept the shares at equal thirds. The γ-factor results at FLD showed less variation (see Fig. 8). This was in fact because the respondents changed only very little of the original ratio, which is 33% of every sustainability sphere (environmental, social, economic). Only FLD 2 changed the ratio to 40% in social and environmental and 20% in the economic dimension.

**Fig. 9:**
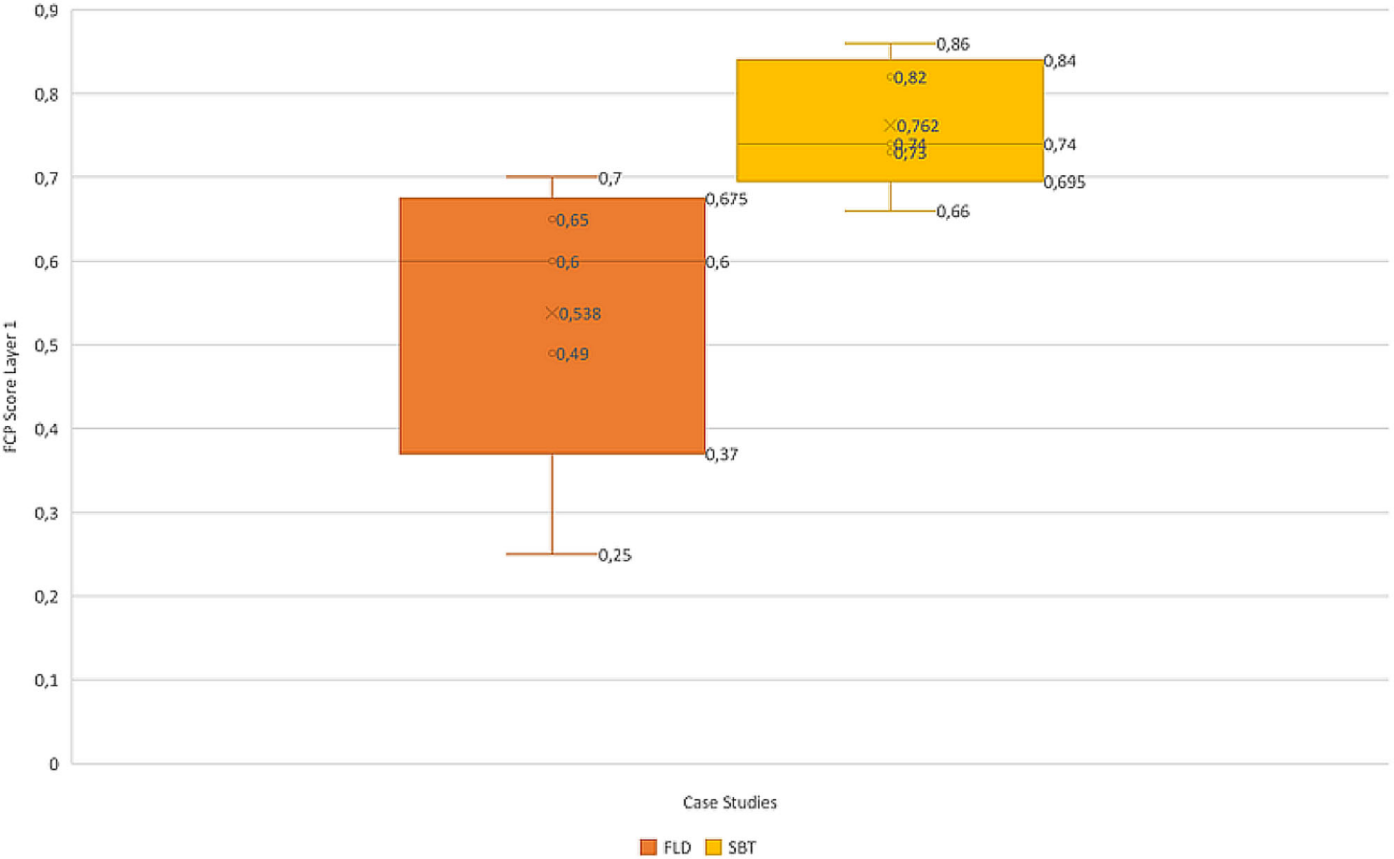
Boxplot of FCP final scores in Layer one at FLD and SBT, with the horizontal line being the median and x the arithmetic mean

The global perspective on how the numerical values differed from SBT case study to FLD, showed a comparably higher score at SBT with 0.77 than at FLD with a score of 0.54 (see Fig. 9). This tendency shows a more positive view, or paraphrased a less negative at the SBT in comparison to the FLD case. As the scores are not the only result in this FCP approach, the numerical values and their differences also show potentially more conflicting points at FLD than at SBT. A comparison between the two case studies is possible, if it is adequate is a matter of discussion in the next section.

Besides the information from the numerical descriptive analysis, the respondents provided an overview of issues with greater individual concern than others by weighting the issues. These individual weightings offer an essential point to find conflicting issues and/or compromise solutions to mitigate difficulties in management early on. These issues can be identified in the final numerical value as well as in the individual ratings. In the FLD, points of conflict could be identified in the environmental indicator group in which the respondents’ ratings did not overlap much (see Fig. 8). Potential for conflicts in environmental realms could be identified within the field of agricultural and touristic use of the area vs. environmental protection efforts. The SBT showed a rather homogeneous weighting in terms of dimensions of sustainability (see Fig. 7). Nevertheless, besides the accreted situation at SBT there was conflict potential between environmental protection and energy production identified. What is being omitted in this results section is the underlying interests, social values, and backgrounds that have a great influence on the individual ratings. For a closer look at the questions/indicators posed, please see the supplementary material. The following section discusses the results of this study with respect to usability and further research needs.

## Discussion

### Subsumption of the Results and Experiences in Using FCP Methodology

The social impacts of reservoirs and dams draw more and more attention, especially since the establishment of the World Commission on Dams (WCD) and their final report in the year 2000 (World Commission on Dams 2000). The research focused on international and very large reservoirs which were built during the time of active work of WCD and in the second half of the 20th century. The dams’ impacts are widely assessed through Social Impact Analysis (SIA) (Égré and Senécal 2003; Kirchherr et al. 2016; Kirchherr and Charles 2016), representing a qualitative way of analyzing the impacts. In this study, we introduced a semi-quantitative way of assessing managerial and social perspectives on large dams in Germany with the FCP methodology. The results represent the individual assessments of the 10 respondents in this survey. A comparison shows great similarities and differences both within the different study areas as well as in the individual ratings. This fact was expectable and the personal tendencies and weights are amongst the central aims of this approach. By analyzing the results from Layer one to Layer four the tendencies and weights in the data sets can offer potentials of deeper analysis irrespective of how varied the respondents’ ratings have been. The rather objective nature of the final numeric values does not mean that these values are in fact objective as it is their individual weight and perception which makes them a promising field of study. All in all, the numerical nature of the results does have advantages over other impact assessments if it comes to comparison of the results. But for a deeper understanding of local implications of management, further studies are needed. The comparison of results is solely possible within data sets of singular cases. Since the questionnaire had to differ in between the case studies, the results can show tendencies, but could not fulfill exact comparisons. Nevertheless, the quick comparison can facilitate transitions of best practice examples and may benefit other case studies.

The fact, that the respondents almost exclusively favored the ecology/environmental sphere in the dimensions of sustainability (γ-factor), revealed a strong connection to the natural environment. Even though a previous study showed economic interests being one of the central points (Daus et al. 2019), these tendencies could not be verified during the evaluation of this survey. This could well be founded in the relatively small number of respondents. Additionally, it could be rooted in the comparably longer history of the SBT (built 1926) (Janzing 2002), which supposedly accreted with the surrounding region and founded a common perceived value of this particular reservoir which is hence seen as being part of the environment (Batel et al. 2013; Zoellner et al. 2008). The FLD on the other hand is rather new and people still remember a time without the existence of the lakes, sometimes even owned land which is now flooded by the reservoirs which creates a strong connection to the “pristine landscapes before the construction of the reservoirs was finished”, which is also observed within review studies (Kelly-Richards et al. 2017). Furthermore, the FLD created a serious touristic infrastructure with connected sources of income for the region, which involves more stakeholders and media attention and their connected agenda setting momentum of the coverage (Eichhorn 2005; Jäckel et al. 2019; McCombs et al. 1972). This made it easier to contact and find experts in the FLD than at the SBT which could play a role if larger samples should be gathered.

The fuzziness of the indicators, e.g., concerning individual understanding and the relatively broad array of the respondents’ professions is a matter of concern given the relatively small size of the sample. With five respondents at each reservoir, the data is of genuine qualitative nature and to be understood as a methodological test or proof of concept rather than a complete and full analysis of each case study. Although the indicators were prepared and tested intensely, there is still never a full proof of 100% appropriateness in the indicators. The effect may be intensified by the “outsiders” view of the researchers in some points, where in others, it may have helped pose neutral/realistic questions (Mayring 2016).

But even with the formulation of fuzzy statements, positive tendencies and conflicting issues could be identified. Within the case study of the FLD conflicting issues arise mostly in between nature protection and tourism/ agriculture. This may not very surprising, but there are compromises on which both sides rely which is mostly valuing the fair management for trying to keep all interests in mind. Within the SBT the conflicting issues were indeed of similar nature, but the spatial focus was extended to the underlying areas of the Murg river as well.

### FCP for Social Ratings of Water Infrastructure Projects?

The examination of responses to infrastructural projects can address a more holistic way of assessing basic infrastructure. This can be understood in a sense that a distinct focus on support, but also opposition, resistance, agreement and participation can impact policy making by displaying an accurate (qualitative) comparable number of approval/disapproval of certain managerial points, which may foster sustainability of reservoir management in the future (Batel et al. 2013). FCP may in this sense be a feedback opportunity for other methodological approaches to gain insights on social perception as in interview and traditional questionnaire approaches (Dopico et al. 2022; Salinas et al. 2019; Pirog et al. 2019; Daus et al. 2019). To put the FCP approach in perspective, it may benefit transdisciplinary research question in which the understanding of objectivist and constructivist understandings come together. Therefore, FCP may be able to bridge a gap of these (sometimes) antithetical worldviews in satisfying numerical and modeling analysis for more technical oriented understandings, while still offer in-depth and perception analysis possibility for social sciences (Haas and Stork 2016). Nevertheless, since it cannot achieve a complete satisfaction of both worlds, further improvements would be desirable. This could comprise open questions for deeper qualitative analysis, to gain insight on the social value and perception influencing the ratings and further possibilities to model and assess numerical values for future scenarios.

While the methodology of FCP was usually used to derive numerous indicator values from existing, built, or planned objects in relation to the research question by the researcher themselves (Freeman 2008), it is herein used by affected people with expert knowledge to rate the indicators, chosen by the researchers, individually. The results, therefore, represent a qualitative overview of the general possibility to grasp a distinct value on different aspects of water related management questions, biased by the setup of the approach. Still the approach could show very individual assessment, which indeed guide towards underlying perceptions.

The results do not describe any sort of representativeness as in surveys, since the knowledge to rate the indicators is quite advanced and could not simply be answered by the general public. The choice for Microsoft Excel (2019) proved functional and useful for the purpose of this study. Nevertheless, other platforms and possibilities should be tested to create easy to understand questionnaires, while still get accurate results.

If compared to standardized quantitative questionnaire methodology (Baur and Blasius 2019) FCP has an advantage of directly pointing out critical solutions, or standpoints that could represent a compromise between differing perspectives and stakeholders as a direct extension of compromise programming based on composite metrics and also used to analyze economic questions (André and Romero 2008). The individual standpoint is a criterion FCP focuses on. It may represent an approachable way for first hints towards conflicts but may be extended by further qualitative and quantitative approaches to deepen first insights.

### FCP as a Semi-Standardized Tool for Evaluation?

Since the questionnaire didn’t include any sort of open question, the numerical values can be compared. Even though the indicator had to differ in total number and specification, the final numerical values can be compared at one glance. This is particularly interesting when going deeper into the evaluation, possibly with further interviews or large-scale questionnaires. The fact that the indicators in question have to be set in advance by the researcher, makes it difficult to standardize the steps, as this will always be up to personal biases of the involved people. Nevertheless, to find the indicators and indicator categories the researcher has to gain in-depth understanding of the study areas. This was achieved here within two preliminary studies (Daus et al. 2019; Daus et al. 2021), which still represent a very individual, or biased, understanding of the matter.

FCP can be seen as a good opportunity for an initial and transdisciplinary method to approach water resources management and other environmental management fields. The numerical nature of the approach and its results offers more technical oriented disciplines a quick overview of transparent ratings and possibility of optimization of larger differences in between the ratings. These possible differences can present an opportunity for social sciences to get deeper into the conflicting issues e.g. with conflict analysis (Haas and Stork 2016).

For methodological improvements, it would be wise to enhance the rating possibilities of respondents further in providing negative rating options for the α-factor ratings. The approach to deal with gaps in the indicators has to be pre-set with every study using directly posed FCP. Depending on the number of gaps (unrated indicators) in the data set, the gap factor could either be eliminated and mean values of the indicator categories can be taken with sporadic gaps. In this study there were considerable gaps in the data set, therefore the gap factor needed to be in place. The decision of using this mathematical approach needs to be set in advance but adjusted individually from case to case. The extreme values in rating gaps, where whole indicator categories are being left out, need to be addressed methodologically to account for imprecise results.

Furthermore, the possibility for the respondents to enhance the base and information depth of the indicators could be a step to fully grasp local and in-depth information. This was initialized through previous interview, media and interdisciplinary reservoir management evaluation (Daus et al. 2019; Daus et al. 2021), but would be more effective in direct questioning despite the implications this would pose concerning the results of questionnaires and potential biases resulting in the presence of the researcher (Kromrey et al. 2016).

The rather positivistic approach in this study can be put into perspective with biases rooting in discursive and post-structural theories concerning reservoir management (Baghel and Nüsser 2010). As researchers/ authors of this study and human beings, we are working with a personally biased version of an impartial truth in the formulation of indicators and the approach in general. Same as biases routing in the group of respondents answering questionnaires in respect to age, gender and professional expertise (Baur and Blasius 2019; Flick et al. 2017; Kromrey et al. 2016; Mattissek et al. 2013). Therefore, the given results always also measure distances from individual worldviews by the researchers and respondents.

The integration of power relation research in social systems would enhance the quality of the FCP approach in empirical social research to point out inequality and power imbalances (Dryzek 2013; Herrera 2018). This would improve the results in integrating perspectives from the discourse analysis field (Waitt 2016), centrally coming from in Foucault’s theories (Diaz-Bone 2006; Foucault and Konersmann 2017; Jäger 2015). This way, the discussed topic of acceptance of management decisions may be expanded by discussions about participation of all stakeholders in management. An implementation of an open question to explain choices may present such a further step for FCP.

The opaque way of responding to the questionnaire via e-mail contains challenges and advantages. Challenging was the fact that it is unclear if the respondents understood the different indicators correctly or rated them just to save their professional reputation. These inaccuracies usually lose in importance and impact with rising numbers of participants (Baur and Blasius 2019). In this study, the sample was comparatively small and was aiming at a general test of the methodological appropriateness of FCP in such a context. The smaller sample size, therefore, shows general possibilities and overall comparability of the approach, with qualitative depth of information enabling the researcher to gain insides (Flick 2017). One advantage is the “objective” answer of the respondents. A questionnaire answered without any sort of personal influence through the researcher will yield more honest and accurate answers (Baur and Blasius 2019).

### Advantages Using FCP

Prediction of outcomes is a crucial point for hydrological management questions. Therefore, the approach in this paper can boost an understanding of social systems to better be able to predict outcomes of management choices (Thompson et al. 2013), without neglecting the integration of empirical social science knowledge. The use of FCP could deliver a tool for such predictions. This could lead to the integration of more social perceptions into frameworks for a sustainable water management in more technical oriented projects (Brown et al. 2015; Maia and Pereira 2015). As it may help to understand the (re)production of knowledge and perception in sustainable water governance (White et al. 2019). Nevertheless, it has to be pointed out that the ranking order of the final results suggests an exact numerical value which is effectively sub-optimal when qualitative indicators are used in FCP, since the numerical values are solely for a first impression. The order of the scores indeed sets the ratings and weights in perspective and qualitative social science and modeling qualities can be brought together to create an interdisciplinary tool to find conflicting issues (Haas and Stork 2016). Interdisciplinary understanding could be supported via a parameterization of personal ratings in FCP which could also lead to an enhanced understanding of human-water interactions as proposed by Vörösmarty et al. (2015). It can also facilitate monitoring and stimulation of society-water interactions by integrating different perspectives and case studies (Ward et al. 2019).

The more conceptualizing findings of this paper could improve sustainable integrated water resources management in perspective of reservoirs (Grigg 2016). Especially knowledge about the perspective on decision making and acceptance/participation in groups of affected people could be improved by this method and it can be used to develop new insights on social questions of water management and renewable energy production on a case study basis.

Still there are some methodological improvements to be made in order to provide more robust results. The questionnaire needs to be more intuitive to achieve a maximum in return rate and avoid comprehension issues. A layout with a plug-and-play attitude for the respondents would potentially generate more answered questionnaires. A simplified mode to rate and individually weight the indicators would possibly lead to less gaps in the data set.

The final result lets the researcher assess the tendency of the respondent at a glance. FCP could bring an advantage compared to classical questionnaire, since personal tendencies and rough evaluation are already integrated as part of the process. Personal weight possibilities outperform classic questionnaires, as their nature may represent the very individual perception and access for case specific management to achieve sustainable solutions. Sustainable approaches in planning and maintaining (critical) infrastructure are a crucial step toward the integration of a just and fair management for all stakeholders involved (Görg et al. 2017).

### New Perspectives on Management Through Use of FCP

The term “sociohydrology” summarizes one of the fields to tackle contemporary and future challenges in water management in relation to societal needs and perceptions to achieve the aims of the United Nations Sustainable Development Goals (SDG) (Di Baldassarre et al. 2019). FCP could lead to a better understanding of “sociohydrologic” questions in the future and can contribute to the successful implementation of the SDGs in the water management sector. Therefore, it could present a solution in helping to solve some of the unsolved problems in hydrology (Blöschl et al. 2019) in respect to water reservoirs and the implementation of sustainable management in the water-energy(-food-health)-nexus.

The direct focus on in-depth conflicting issues and possible compromise solutions offers a practical way of organizing fuzzy sets in social and managerial research. This could possibly help mitigating conflicts between different stakeholders and their interests in (infrastructure/water) projects from early on. If bigger data sets are getting collected and the case studies are extended to internationally recognized and researched dams and their reservoirs, an evaluation of the quality of FCP in water management questions in comparison to more established methodology, e.g. SIA, would become possible. Perceptions and management decisions can be understood in more detail with interview techniques (Pirog et al. 2019; Dopico et al. 2022). Still, FCP could improve management related challenges concerning societal issues, which could help preparing for a more sustainable process in the face of climate change on an international scale (Cherry et al. 2017; Jamali et al. 2013). FCP could represent a way to initially approach these issues from an interdisciplinary perspective and offer first insight on social perceptions of management.

## Conclusion and Outlook

This study tried to shed light on a rather technical methodology to find answers, priorities, conflicting issues and compromise solutions in complex managerial interactions for social interests in reservoir bound questions. The idea was to provide a questionnaire to be rated personally by experts in different aspects of singular indicators (statements), indicator categories, compensational factors, and dimensions of sustainability to derive a final single numerical value.

FCP could provide a comparable score on approval/disapproval and critical/trade-off issues of a project which concerns social systems in the perimeter. With the FCP methodology, all numerical values rated are being summarized to one final score (with 1 being the absolute optimum and 0 being the inferior value), without losing any information on how the score was generated when retrieving to the single indicators. Therefore, the methodology could well fit comparable initial qualitative research in the field of questioning experts. In this study, water management and specifically reservoir management perceptions were asked about at two case studies in Germany. The results in this survey showed a clear tendency towards more conflicting issues at a rather new reservoir FLD with an overall score of 0.54 compared to the more established one SBT 0.77.

Since the FCP method as such was never used in direct questioning of affected people in this realm, this study proved the general possibility and feasibility, with further improvements and adjustments still to be undertaken. Analysis of power relations and differing political clouts would deepen the understanding of local conditions with FCP. The results are based on a relatively small number of participants, since the knowledge base to rate the indicators has to be quite elaborate. There is still the need for more and larger samplings with FCP in the realm of (qualitative) socio-economic research with environmental and managerial implications and water-based questions to deepen and advance the experience and use in this field. The mathematical process and computer program in question could still be improved/simplified, but provided a solid way of calculation. Indeed, the FCP could enhance directly obtained social perceptions, start active debates and therefore help the management of environmental-social systems to find solutions for all stakeholders involved and generate more just decisions.

## Supplementary Information

Supplementary Material

## Data Availability

See https://github.com/milan-daus/Fuzzy-Composite-Programming-approach to access the questionnaire with the mathematical approach.

## References

[CR1] Achatz R, Kamuf I (2016) Unterirdisches Speicherbecken für das Pumpspeicherkraft-werk Forbach. Wasserwirtschaft (6). https://www.enbw.com/media/kon-zern/docs/energieerzeugung/fachaufsatz-wasserwirtschaft-unterirdisches-speicherbecken-fuer-das-pumpspeicherkraftwerk-forbach.pdf

[CR2] André FJ, Romero C (2008). Computing compromise solutions: On the connections between compromise programming and composite programming. Appl Math Comput.

[CR3] Ansar A, Flyvbjerg B, Budzier A, Lunn D (2014). Should we build more large dams? The actual costs of hydropower megaproject development. Energy Policy.

[CR4] Arunraj NS, Maiti J (2009). Development of environmental consequence index (ECI) using fuzzy composite programming. J Hazard Mater.

[CR5] Awojobi O, Jenkins GP (2015). Were the hydro dams financed by the World Bank from 1976 to 2005 worthwhile. Energy Policy.

[CR6] Baghel R, Nüsser M (2010). Discussing large dams in asia after the world commission on dams: is a political ecology approach the way forward?. Water Altern.

[CR7] Bai X, van der Leeuw S, O'Brien K, Berkhout F, Biermann F, Brondizio ES, Cudennec C, Dearing J, Duraiappah A, Glaser M, Revkin A, Steffen W, Syvitski J (2016). Plausible and desirable futures in the Anthropocene: A new research agenda.. Glob Environ Change.

[CR8] Bardossy A, Duckstein L, Bogardi I (1993). Combination of fuzzy numbers representing expert opinions.. Fuzzy Sets Syst.

[CR9] Bárdossy A, Bogárdi I, Duckstein L (1985) Composite programming as an extension of compromise programming. In Serafini P (Ed.), Mathematics of Multi Objective Optimization (pp. 375–408). Springer Vienna. 10.1007/978-3-7091-2822-0_15

[CR10] Bárdossy A, Duckstein L (1992). Analysis of a karstic aquifer management problem by fuzzy composite programming.. J Am Water Resour Assoc.

[CR11] Bàrdossy A, Bogàrdi I, Duckstein L, Nachtnebel HP (1984). Fuzzy decision making in regional water management.. IFAC Proc.

[CR12] Barrow CJ (2010). How is environmental conflict addressed by SIA. Environ Impact Assess Rev.

[CR13] Batel S, Devine-Wright P, Tangeland T (2013). Social acceptance of low carbon energy and associated infrastructures: A critical discussion. Energy Policy.

[CR14] Baur N, Blasius J (Eds.) (2019) Handbuch Methoden der empirischen Sozialforschung (2., vollständig überarbeitete und erweiterte Auflage). Springer Fachmedien Wiesbaden GmbH.

[CR15] Blöschl G, Bierkens MF, Chambel A, Cudennec C, Destouni G, Fiori A, Kirchner JW, McDonnell JJ, Savenije HH, Sivapalan M, Stumpp C, Toth E, Volpi E, Carr G, Lupton CEA (2019). Twenty-three unsolved problems in hydrology (UPH) ‐ a community perspective.. Hydrological Sci J.

[CR16] Bogardi I, Bárdossy A, Duckstein L (1983). Regional management of an aquifer for mining under fuzzy environmental objectives.. Water Resour Res.

[CR17] Briemle K (1996) Das “Neue Fränkische Seenland”: Eine Landschaft aus Menschenhand = Le “nouveau pays des lacs de Franconie”: un paysage façonné par la main de l’homme = The “New Franconian Lake District”: a landscape created by human hand. 10.5169/SEALS-137809

[CR18] Brown CM, Lund JR, Cai X, Reed PM, Zagona EA, Ostfeld A, Hall J, Characklis GW, Yu W, Brekke L (2015). The future of water resources systems analysis: Toward a scientific framework for sustainable water management. Water Resour Res.

[CR19] Brüggemeier F‑J, Rommelspacher T (Eds.) (1989) Besiegte Natur: Geschichte der Umwelt im 19. und 20. Jahrhundert (Orig.-Ausg., 2. Aufl.). Beck. http://www.bsz-bw.de/cgi-bin/ekz.cgi?SWB01840819

[CR20] Carr G, Barendrecht MH, Debevec L, Kuil L, Blöschl G (2020). People and water: understanding integrated systems needs integrated approaches.. J Water Supply: Res Technol-Aqua.

[CR21] Carter J, Chiclana F, Khuman AS, Chen T (2021) Fuzzy Logic. Springer International Publishing. 10.1007/978-3-030-66474-9

[CR22] Cherry JE, Knapp C, Trainor S, Ray AJ, Tedesche M, Walker S (2017). Planning for climate change impacts on hydropower in the Far North. Hydrol Earth Syst Sci.

[CR23] Cinelli M, Coles SR, Kirwan K (2014). Analysis of the potentials of multi criteria decision analysis methods to conduct sustainability assessment. Ecol Indic.

[CR24] Crutzen PJ (2002). Geology of mankind. Nature.

[CR25] Crutzen PJ, Stoermer, EF (2000) The “Anthropocene” (IGBP Newsletter No. 41). IGBP.

[CR26] Daus M, Koberger K, Gnutzmann N, Hertrich T, Glaser R (2019). Transferring water while transforming landscape: new societal implications, perceptions and challenges of management in the reservoir system franconian lake district. Water.

[CR27] Daus M, Koberger K, Koca K, Beckers F, Encinas Fernández J, Weisbrod B, Dietrich D, Gerbersdorf SU, Glaser R, Haun S, Hofmann H, Martin-Creuzburg D, Peeters F, Wieprecht S (2021). Interdisciplinary reservoir management—a tool for sustainable water resources management. Sustainability.

[CR28] Di Baldassarre G, Sivapalan M, Rusca M, Cudennec C, Garcia M, Kreibich H, Konar M, Mondino E, Mård J, Pande S, Sanderson MR, Tian F, Viglione A, Wei J, Wei Y, Yu DJ, Srinivasan V, Blöschl G (2019). Sociohydrology: scientific challenges in addressing the sustainable development goals.. Water Resour Res.

[CR29] Diaz-Bone R (2006) Kritische Diskursanalyse: Zur Ausarbeitung einer problembezogenen Diskursanalyse im Anschluss an Foucault. Siegfried Jäger im Gespräch mit Rainer Diaz-Bone. Forum Qualitative Sozialforschung/Forum: Qualitative Social Research, *7*(3). http://nbn-resolving.de/urn:nbn:de:0114-fqs0603219

[CR30] Dopico E, Arboleya E, Fernandez S, Borrell Y, Consuegra S, Leaniz C, Lázaro G, Rodriguez C, Garcia-Vazquez E (2022). Water security determines social attitudes about dams and reservoirs in South Europe.. Sci Rep.

[CR31] Dryzek JS (2013) The politics of the earth: Environmental discourses (3. ed.). Oxford Univ. Press.

[CR32] Égré D, Senécal P (2003). Social impact assessments of large dams throughout the world: Lessons learned over two decades. Impact Assess Proj Apprais.

[CR33] Eichhorn W (2005) Agenda Setting Prozesse: Eine theoretische Analyse individueller und gesellschaftlicher Themenstrukturierung. München. http://epub.ub.uni-muenchen.de/archive/00000734/

[CR34] EnBW. (2018) Baustein für die Energiezukunft: Das Ausbauprojekt Pumpspeicherkraftwerk Forbach. EnBW. https://www.enbw.com/media/konzern/docs/energieerzeu-gung/flyer-ausbau-pumpspeicherkraftwerk-forbach.pdf

[CR35] Esteban Indurain, Javier Fernandez, & Humberto Bustince (Eds.) (2018) New trends in fuzzy set theory and related items. MDPI. 10.3390/books978-3-03897-124-5

[CR36] Faria F, Davis A, Severnini E, Jaramillo P (2017). The local socio-economic impacts of large hydropower plant development in a developing country.. Energy Econ.

[CR37] Flick U (2017) Qualitative Sozialforschung: Eine Einführung (Originalausgabe, 8. Auflage). Rororo Rowohlts Enzyklopädie: Vol. 55694. rowohlts enzyklopädie im Rowohlt Taschenbuch Verlag.

[CR38] Flick U, Kardorff EV, Steinke I (Eds.) (2017) Rororo Rowohlts Enzyklopädie: Vol. 55628. Qualitative Forschung: Ein Handbuch (12. Auflage, Originalausgabe). rowohlts enzyklopädie im Rowohlt Taschenbuch Verlag.

[CR39] Föhl A, Hamm M (1985) Die Industriegeschichte des Wassers: Transport, Energie, Versorgung. VDI-Verl.

[CR40] Foucault M, Konersmann R (2017) Die Ordnung des Diskurses ((W. Seitter, Trans.)) (Erweiterte Ausgabe, 14. Auflage). Fischer Taschenbuch Fischer Wissenschaft: Vol. 10083. FISCHER Taschenbuch.

[CR41] Freeman B (2008) Modernization Criteria Assessment for Water Resources Planning [Univ. Diss.]. Institut für Wasserbau Universität Stuttgart, Stuttgart.

[CR42] Gonzalez JM, Tomlinson JE, Harou JJ, Martínez Ceseña EA, Panteli M, Bottacin-Busolin A, Hurford A, Olivares MA, Siddiqui A, Erfani T, Strzepek KM, Mancarella P, Mutale J, Obuobie E, Seid AH, Ya AZ (2020). Spatial and sectoral benefit distribution in water-energy system design. Appl Energy.

[CR43] Görg C, Brand U, Haberl H, Hummel D, Jahn T, Liehr S (2017). Challenges for social-ecological transformations: contributions from social and political ecology. Sustainability.

[CR44] Greco S, Ehrgott M, Figueira JR (Eds.) (2016) International series in operations research & management science: Vol. 233. Multiple criteria decision analysis: State of the art surveys (Second edition). Springer. http://ebooks.ciando.com/book/index.cfm/bok_id/199001510.1007/978-1-4939-3094-4

[CR45] Grigg NS (2016) Integrated Water Resource Management: An Interdisciplinary Approach. Springer eBook Collection Earth and Environmental Science. Palgrave Macmillan. 10.1057/978-1-137-57615-6

[CR46] Grun L, Bayerischer Rundfunk. (2021, May 31) Pfofelder sagen Nein zu Center Parcs am Brombachsee [Press release]. https://www.br.de/nachrichten/bayern/pfofelder-sagen-nein-zu-center-parcs-am-brombachsee,SYuKloU

[CR47] Haas T, Stork K (2016) Hochwasser - Schutz - Konflikte: eine transdisziplinäre Perspektive. Universitätsverlag Winter.

[CR48] Herrera RJ (2018) Die Politische Ökologie als Instrument zur Analyse der Regulation von Wassernutzungen: Das Beispiel des oberen Río Negro im Norden Patagoniens, Argentinien [PhD]. KIT, Karlsruhe.

[CR49] Jäckel M, Fröhlich G, Röder, D (2019) Medienwirkungen kompakt. Springer Fachmedien Wiesbaden. 10.1007/978-3-658-24817-8

[CR50] Jäger S (2015) Kritische Diskursanalyse: Eine Einführung (7., vollständig überarbeitete Auflage). Edition DISS: Bd. 3. Unrast.

[CR51] Jamali S, Abrishamchi A, Madani K (2013). Climate change and hydropower planning in the middle east: implications for Iran’s Karkheh Hydropower Systems. J Energy Eng.

[CR52] Janzing B (2002) Baden unter Strom: Eine Regionalgeschichte der Elektrifizierung; von der Wasserkraft ins Solarzeitalter. Dold.

[CR53] Jones D, Barnes E (2000). Fuzzy composite programming to combine remote sensing and crop models for decision support in precision crop management. Agric Syst.

[CR54] Keller D (2014) “Weiße Kohle” im Murgtal. Das Rudolf-Fettweis-Werk in Forbach – eines der ersten Pumpspeicherkraftwerke Europas. 10.11588/nbdpfbw.2012.3.12402

[CR55] Kelly-Richards S, Silber-Coats N, Crootof A, Tecklin D, Bauer C (2017). Governing the transition to renewable energy: A review of impacts and policy issues in the small hydropower boom. Energy Policy.

[CR56] Green K, Armstrong SJ, Graefe A (2007). Methods to Elicit Forecasts from Groups: Delphi and Prediction Markets Compared. Foresight: Int J Appl Forecast.

[CR57] Kirchherr J, Charles KJ (2016). The social impacts of dams: A new framework for scholarly analysis. Environ Impact Assess Rev.

[CR58] Kirchherr J, Pohlner H, Charles KJ (2016). Cleaning up the big muddy: A meta-synthesis of the research on the social impact of dams. Environ Impact Assess Rev.

[CR59] Köngeter J (Ed.) (2013) Talsperren in Deutschland. Springer Vieweg.

[CR60] Kromrey H, Roose J, Strübing J (2016) Empirische Sozialforschung: Modelle und Methoden der standardisierten Datenerhebung und Datenauswertung mit Annotationen aus qualitativ-interpretativer Perspektive (13., völlig überarbeitete Auflage). UTB: Vol. 8681. UVK; UVK/Lucius. http://www.utb-studi-e-book.de/9783838586816

[CR61] Kühne O, Duttmann R (2019) Recent Challenges of the Ecosystems Services Approach from an Interdisciplinary Point of View. Raumforschung Und Raumordnung Spatial Research and Planning, 0(0). 10.2478/rara-2019-0055

[CR62] Lee YW, Bogardi I, Stansbury J (1991). Fuzzy decision making in dredged‐material management. J Environ Eng.

[CR63] Linton J (2014). Modern water and its discontents: a history of hydrosocial renewal. Wiley Interdiscip Rev: Water.

[CR64] Liu J, Yang H, Cudennec C, Gain AK, Hoff H, Lawford R, Qi J, Strasser Lde, Yillia PT, Zheng C (2017). Challenges in operationalizing the water–energy–food nexus.. Hydrological Sci J.

[CR65] Maia R, Pereira LS (2015). Water resources management in an interdisciplinary and changing context. Water Resour Manag.

[CR66] Mattissek A, Pfaffenbach C, & Reuber P (2013) Methoden der empirischen Humangeographie (2. Auflage, Neubearbeitung). Das Geographische Seminar. Westermann.

[CR67] Mayer HO (2013) Interview und schriftliche Befragung: Grundlagen und Methoden empirischer Sozialforschung (6., überarbeitete Auflage).

[CR68] Mayring P (2016) Einführung in die qualitative Sozialforschung: Eine Anleitung zu qualitativem Denken (6., überarbeitete Auflage). Beltz. http://content-select.com/index.php?id=bib_view&ean=9783407294524

[CR69] McCombs, Maxwell E, Shaw Donald L (1972). The agenda-setting function of mass media. Public Opin Q.

[CR70] Pahl-Wostl C (2015) Water Governance in the Face of Global Change: From Understanding to Transformation (1st ed. 2015). Water Governance - Concepts, Methods, and Practice. Springer. http://search.ebscohost.com/login.aspx?direct=true&scope=site&db=nlebk&AN=106062410.1007/978-3-319-21855-7

[CR71] Pirog D, Fidelus-Orzechowska J, Wiejaczka L, Lajczak A (2019). Hierarchy of factors affecting the social perception of dam reservoirs. Environ Impact Assess Rev.

[CR72] Polatidis H, Haralambopoulos DA, Munda G, Vreeker R (2006). Selecting an appropriate multi-criteria decision analysis technique for renewable energy planning. Energy Sources, Part B: Econ, Plan, Policy.

[CR73] Roszkowska E, Kacprzak D (2016). The fuzzy saw and fuzzy TOPSIS procedures based on ordered fuzzy numbers.. Inf Sci.

[CR75] Salinas C, Oliveira V, Brito L, Ferreira A, Araujo J (2019). Social impacts of a large-dam construction: the case of Castanhao, Brazil. Water Int.

[CR76] Savenije H, van der Zaag P (2008). Integrated water resources management: Concepts and issues. Phys Chem Earth, Parts A/B/C.

[CR77] Sivapalan M, Blöschl G (2017). The growth of hydrological understanding: technologies, ideas, and societal needs shape the field. Water Resour Res.

[CR78] Sovacool BK, Walter G (2018). Major hydropower states, sustainable development, and energy security: Insights from a preliminary cross-comparative assessment. Energy.

[CR79] Strobl E, Strobl RO (2011). The distributional impact of large dams. Evidence from cropland productivity in Africa. J Dev Econ.

[CR80] Tabi A, Wüstenhagen R (2017). Keep it local and fish-friendly: Social acceptance of hydropower projects in Switzerland. Renew Sustain Energy Rev.

[CR81] Thompson SE, Sivapalan M, Harman CJ, Srinivasan V, Hipsey MR, Reed P, Montanari A, Blöschl G (2013). Developing predictive insight into changing water systems: use-inspired hydrologic science for the Anthropocene.. Hydrol Earth Syst Sci.

[CR82] Vörösmarty CJ, Meybeck M, Pastore CL (2015). Impair-then-repair: a brief history & global-scale hypothesis regarding human-water interactions in the anthropocene. Daedalus.

[CR83] Vorosmarty C, Lettenmaier D, Leveque C, Meybeck M (2004). Humans transforming the global water system. Eos, Trans Am Geophys Union.

[CR84] Waitt GR (2016) Doing Dicourse Analysis. In I. Hay (Ed.), Qualitative research methods in human geography (pp. 163–191). Oxford University Press.

[CR85] Ward NK, Fitchett L, Hart JA, Shu L, Stachelek J, Weng W, Zhang Y, Dugan H, Hetherington A, Boyle K, Carey CC, Cobourn KM, Hanson PC, Kemanian AR, Sorice MG, Weathers KC (2019). Integrating fast and slow processes is essential for simulating human-freshwater interactions. Ambio.

[CR86] Weber D (2016) Composite Programming als Methode der politisch-geographischen Konfliktanalyse: Vergleich, Optimierung und digitale Transformation [Bachelor Thesis [unpublished]]. Albert-Ludwigs-University, Freiburg im Breisgau.

[CR87] White DD, Lawless KL, Vivoni ER, Mascaro G, Pahle R, Kumar I, Coli P, Castillo RM, Moreda F, Asfora M (2019). Co-producing interdisciplinary knowledge and action for sustainable water governance: lessons from the development of a water resources decision support system in Pernambuco, Brazil. Glob Chall (Hoboken, NJ).

[CR88] Wilkens I (2012) Multikriterielle Analyse zur Nachhaltigkeitsbewertung von Energiesystemen – Von der Theorie zur praktischen Anwendung [Dissertation]. Technische Universität Berlin, Berlin. https://d-nb.info/1027798160/34

[CR89] World Commission on Dams (2000) Dams and development: A new framework for decision-making. Earthscan.

[CR90] World Energy Council (Ed.) (2016) World Energy Resources: Report 2016.

[CR91] Wüstenhagen R, Wolsink M, Bürer MJ (2007). Social acceptance of renewable energy innovation: An introduction to the concept. Energy Policy.

[CR92] Zadeh LA (1965). Fuzzy sets. Inf Control.

[CR93] Zarfl C, Lumsdon AE, Berlekamp J, Tydecks L, Tockner K (2015). A global boom in hydropower dam construction. Aquat Sci.

[CR94] Zoellner J, Schweizer-Ries P, Wemheuer C (2008). Public acceptance of renewable energies: Results from case studies in Germany. Energy Policy.

