# Supplementary material for: Application of Fuzzy Composite Programming in a Questionnaire as a Methodological Test to Study the Effect of Reservoir Management on Social Interests—A Survey Based on Two Case Studies in Southern Germany
Source: Environ Manage. 2023 Feb 11;71(6):1145–61. doi: 10.1007/s00267-023-01799-9 (PMC10183413; doi:10.1007/s00267-023-01799-9)
Supplement: Supplementary file 1 — Supplementary Material [file 267_2023_1799_MOESM1_ESM.pdf]

Supplementary Material

Table 1: Indicators for the experts at the FLD (translated from German, therefore the original meaning could have been changed slightly)

Legislation, administration, construction and operation
The construction of the dams and the damming of the lakes followed formal legal guidelines.
The construction of the lakes went smoothly.
Different interests were considered during construction.
The takeover of the land by the Free State of Bavaria went smoothly.
The legal requirements for operation were/are met.
The monitoring carried out (safety, quality assurance) complies with the legal requirements.
In the case of costs for operation and possible subsequent financial burdens, it is clearly regulated who will take care of them.
The interests of groups/companies/institutions are impaired during operation or are subordinated to the project.
Different interests are considered during operation.
The water transfer has negative impacts on existing agreements, e.g. Water Framework Directive, or Natura 2000.
Water use rights have developed positively as a result of the project in the immediate vicinity.
Environmental factors
During construction there have been adverse impacts, e.g. on landscape or nature conservation areas, which are still relevant today.
The development of the FLD has had a negative impact on vegetation, such as e.g. meadow areas.
During operation, there are adverse impacts on landscape or nature conservation areas.
The original flora and fauna in the region of the FLD has been negatively impaired by the construction of the lakes.
The population is burdened by the existence of the breeding and feeding areas of (water)birds.
The bird island in the Lake Altmuehl is a very positive aspect of the FLD.
The flow behaviour of the Altmuehl river was and is negatively influenced by the FSL.
A deterioration of water quality is to be feared due to nutrient inputs.
Water quality is deteriorating due to the construction of the lakes.
Agriculture is causally involved in the deterioration of water quality in the lakes.
The sewage treatment plants in the upper reaches of the Altmuehl river and in the region are causally involved in the deterioration of the water quality of the lakes.
Blue-green algae (cyanobacteria) are a major problem for the lakes.
Invasive species such as triangular mussels are a major problem for biodiversity.
White fish are a major problem for biodiversity in the lakes of the FLD.
Wild geese are a major problem in the FLD.
There are noticeably more mosquitoes in the region due to the lakes.
Economic factors
The development of the FLD has had positive impacts on the economic performance of the region.
The development of the FLD has positive impacts on the economic performance of the region.
The development of the FLD will have a positive impact on the region's economic performance.
Measures are needed to ensure that the region's economic performance is not jeopardised (e.g. tourism).
Appropriate efforts will be made to exploit new tourism potentials.
The image as a tourism region is further enhanced by the lakes.
Water transfer has proven to be very positive in the Rhine-Main region (Rezat, Roth, Rednitz, Main, Rhine).
The running costs of the measure remain within reasonable limits.
The costs of operating the transfer are justifiable for the resulting benefits.
The costs of operating the infrastructure are also justified by the income from the tourism industry.

The agricultural use of the adjacent areas is made more difficult.
Possible costs due to damage to adjacent infrastructure (e.g. sewage system, cycle paths, roads, beach areas) are sufficiently considered.
Risk factors
All (safety) objections and concerns have been and will be dealt with appropriately.
Dike and embankment maintenance are ensured and sufficient.
There is an increased risk for local residents.
Citizens are burdened by the risk of rising groundwater levels.
Sufficient measures are taken to inform the population about possible risks.
Blue-green algae (cyanobacteria) pose a health risk.
Risk concerns of local residents are taken seriously and dealt with.
Potential investors are deterred by existing risks.
The facilities are resistant to extreme high or low water influences.
Facilities are vulnerable to external disturbances such as earthquakes, drought or continuous rain.
Technical factors
Proven technologies are used.
Pressure water (increased groundwater level) poses a problem for the use of surrounding land.
Usable land and residential areas in the vicinity of water bodies are negatively influenced by fluctuating water levels (large Lake Brombach).
The water transfer worsens the flow behaviour of the inflows and outflows.
The water transfer through Lakes Altmuehl and Brombach functions very well.
The design basis (precipitation amounts) for the construction of the facilities is still sufficient.
There is sufficient monitoring of the technical components of the reservoir system.
The costs for monitoring water quality and safety aspects are reasonable and appropriate.
The continuous operation, maintenance and care of the facilities is guaranteed.
Siltation of the Lake Altmuehl due to sediments is adequately counteracted.
Benefits / impacts of the facility
The overall benefit of the FLD can be rated as positive.
The function of the lakes in the event of flooding is positive.
The purpose of the lakes to raise the low water level is fulfilled.
The downstream riparians on the Altmuehl river benefit from the flood protection provided by the facilities.
The positive aspects of the lakes outweigh the negative aspects.
Greenhouse gases such as methane and CO_2 are a major problem at the FLD.
Biofilms (slime layer of microorganisms, bacteria, fungi & algae) are a big problem in the FSL's lakes.
(Blue) algal blooms (cyanobacteria blooms) are a big problem at the FLD's lakes.
The water quality of the lakes sometimes poses a health risk to people.
Governance/ Management
Stakeholder objections and concerns are responded to appropriately.
There are management contacts for concerns about the lakes.
Sufficient management strategies are in place to satisfy the various user demands.
The management of the lakes is carried out in a comprehensible manner.
The management manages the challenges on the lakes very satisfactorily.
There are winners in all areas of the FLD.
There are losers in all areas of the FLD.
Better management would contribute to a positive coexistence of nature conservation, tourism, agriculture and local residents.
The municipalities involved (Zweckverbaende) etc. are also financially involved according to their limitations and opportunities, or there is compensation in other ways.
There is fairness and willingness to talk about all current developments concerning water management.

The influence of nutrients on the water quality of the lakes is sufficiently counteracted.
The existing problem of blue-green algae (cyanobacteria) is responded to appropriately and possible solutions are presented.
The existing wild goose population is dealt with appropriately.
Overall, the water management authority handles the challenges at the Franconian Lake District very satisfactorily.
An exchange of knowledge with similar projects takes place.
Social interests
Uses other than water management are often restricted.
Measures are necessary in order not to endanger the quality of local recreation.
The FLD has been well designed to meet all demands.
Local residents are not disadvantaged by the marketing of the FLD as a tourism region.
The development into a tourism region is positive.
The region as a whole has benefited greatly from the construction of the lakes.
The region as a whole has suffered greatly from the construction of the lakes.
Sufficient attention is paid to tourism interests.
Sufficient attention is paid to water management requirements.
Sufficient attention is paid to flood protection.
Sufficient attention is paid to nature conservation interests.
Sufficient attention is paid to the interests of local residents.
Infrastructure and traffic
The project has a negative impact on traffic volume.
The road infrastructure lost due to the flooding of the lakes could be usefully built elsewhere.
The newly created infrastructure meets its requirements.
The roads for the cultivation of agricultural land can be used without restrictions.
Public transport has developed positively.
The infrastructure at the lakes (e.g. cycle paths, public toilets, restaurants) has developed positively.
The lake centres are well developed by the Zweckverbaende.
The infrastructure at the lakes also benefits the local population.

Table 2: Indicators for the experts at the SBT (translated from German, therefore the original meaning could have been changed slightly

Legislation, administration, construction and operation
The legal requirements for operation have been and will be complied with.
The project has negative impacts on existing national and international agreements, e.g. Water Framework Directive, or Natura 2000.
The (further) development of the dam complies with the legal requirements.
The monitoring carried out (safety, quality assurance) complies with the legal requirements.
If costs arise for the operation of the spillway and possible measures, it is determined who will bear them.
Groups/companies/institutions are impaired in their interests during operation or are subordinated to the project.
Different interests are considered in the expansion of the dam.
Different interests are considered during operation.
Water use rights have been negatively affected by the project in the surrounding area.
Environmental factors
(Blue) algae (cyanobacteria) are a problem for the uses at the dam.
The construction of the dam has negatively affected vegetation such as meadow areas.
During operation, there are adverse effects on landscape or nature conservation areas.

The original flora and fauna in the region have been negatively affected by the construction of the dam.
The flow behaviour of the Schwarzenbach-stream has been and will be negatively affected by the dam.
The water quality in the dam is deteriorating due to nutrient inputs.
Water quality deteriorates due to silting in the dam.
Water quality deteriorates due to the use of the dam as a pumped storage plant.
The planned expansion and renewal of the dam (Streitmannskopf extension, cavern power plant) causes conflicts with the National Park.
The planned expansion and renewal of the dam (Streitmannskopf expansion, cavern power plant) takes sufficient account of environmental concerns.
Economic factors
The ecological benefits of the planned fish lift on the river Murg in Forbach are in proportion to its costs.
The use as forestry lands of the adjacent areas at the dam is made more difficult.
There are economic sectors that are currently negatively affected by the dam.
Damage is caused to local residents by the use of the dam.
The dam is economic valuable for the region as a whole.
The image as a tourist region is enhanced by the dam.
Surrounding businesses are not disadvantaged by the marketing of the dam as a tourist region.
Damage can be caused to local residents by the expansion of the dam.
Appropriate efforts are made to exploit new tourism potential.
The dam will be economically viable for the region in the future.
Risk factors
All (safety) objections and concerns have been and will be dealt with appropriately.
The maintenance of the dam is guaranteed and sufficient.
There is an increased risk to residents from the dam.
Risk concerns of local residents are taken seriously and addressed.
Sufficient measures are taken to inform the population about possible risks.
Potential investors are deterred by existing risks.
The plant is resistant to extreme high or low water influences.
The plant is vulnerable to external disturbances such as a severe flood, earthquake or continuous rain.
The facility is technically vulnerable to unexpected changes in the natural inflows of the Schwarzenbach-stream.
Technical factors
Pressure water poses a problem for the use of surrounding land.
Usable land and residential areas in the vicinity of the dam are negatively affected by fluctuating water levels.
Water retention significantly reduces runoff in the lower reaches.
The design basis (precipitation amounts) for the construction of the dam is still sufficient today.
There is sufficient monitoring of the technical components of the dam.
The monitoring effort is justifiable.
Continuous operation, maintenance and care of the facility are guaranteed.
Siltation of the dam due to sediments is adequately counteracted.
The technical components of the dam will meet future requirements.
Benefits / impacts of the facility
The overall benefit of the Schwarzenbach-dam can be rated as positive.
The impacts of the dam in the event of flooding are positive.
The impacts of the dam in the event of flooding are negative.
The downstream riparians on the Schwarzenbach benefit from the flood protection provided by the facilities.

Greenhouse gases such as methane and CO_2 are a major problem at the dam.
Biofilms (slimy layer of microorganisms, bacteria, fungi & algae) are a problem in the dam.
Blue algal blooms (cyanobacteria) are a problem at the dam.
The water quality of the dam is partly a health risk for people.
The facility contributes significantly to renewable energy generation.
Governance / Management
Stakeholder objections and concerns are responded to appropriately.
There are management contacts for concerns about the dam.
There are sufficient management strategies in place to satisfy the various user demands.
The management of the dam is carried out in a comprehensible manner.
There are winners in all areas of the dam.
There are losers in all areas of the dam.
Better management of risks would lead to an increase in investment in the region.
Those who are disadvantaged are sufficiently compensated for it (e.g. industry).
The municipalities etc. involved are also financially involved according to their restrictions and opportunities, or there is compensation in some other way.
There is fairness and willingness to talk about current developments in water management.
The existing problem of blue-green algae (cyanobacteria) is responded to appropriately and possible solutions are presented.
EnBW (owner) handles the challenges at the Schwarzenbach-dam satisfactorily overall.
Efforts are being made to exchange knowledge with other dams.
The negative influence of the introduced nutrients on the water quality is sufficiently counteracted.
Social interests
Uses other than water management are limited.
Given the current development, measures are necessary to ensure that the quality of local recreation is not jeopardised.
The Schwarzenbach reservoir is well designed to meet all requirements.
Local residents are not disadvantaged by the marketing of the dam as a tourist region.
The development into a tourism region is positive.
The region as a whole has benefited greatly from the construction of the dam.
The region as a whole has suffered greatly from the construction of the dam.
The region as a whole will benefit greatly from the development of the dam.
Sufficient attention is paid to tourism interests.
Water management requirements are sufficiently considered.
Sufficient attention is paid to flood protection.
Sufficient attention is paid to nature conservation interests.
Sufficient attention is paid to the interests of local residents.
Infrastructure and traffic
The project has a negative impact on traffic volume.
The infrastructure at the dam also benefits the local population.
The current infrastructure meets their needs.
Public transport has developed positively.
The infrastructure at the dam (e.g. cycle paths, public toilets, restaurants) has developed positively.
